# Mesenteric root pseudocyst: finding in an asymptomatic patient—a case report

**DOI:** 10.1186/s40792-024-01830-z

**Published:** 2024-03-29

**Authors:** F. Suldrup, P. Uad, A. Vaccaro, M. Mazza, J. Santino, O. Mazza

**Affiliations:** Hospital Italiano de Buenos Aires, Ciudad Autónoma de Buenos Aires, Argentina

**Keywords:** Mesenteric cysts, Pseudocyst of the mesentery, Abdominal mass

## Abstract

**Background:**

Mesenteric cysts are one of the rarest abdominal tumor masses, representing a little-studied pathology. In turn, the variability and non-specificity of clinical manifestations make diagnosis difficult, as it can be reached by imaging findings due to another cause or by non-specific abdominal pain.

**Case presentation:**

This article describes the case report of an asymptomatic 28-year-old patient who presented a 6-cm abdominal cystic mass with mixed density, which was found incidentally by computed tomography. Exploratory laparoscopy was performed followed by conversion to conventional surgery to extract the tumor mass. The anatomical pathology diagnosis was pseudocyst of the mesentery root. Mesenteric cysts are one of the rarest abdominal tumor masses, representing a little-studied pathology. In turn, the variability and non-specificity of clinical manifestations make diagnosis difficult, as it can be reached by imaging findings due to another cause or by non-specific abdominal pain.

**Conclusions:**

Mesenteric cysts are rare, and their nonspecific symptoms often lead to diagnosis based on imaging findings. Complete laparoscopic enucleation is the standard treatment.

## Introduction

Mesenteric cysts are one of the rarest abdominal tumor masses; approximately 820 cases have been reported since 1507 [1], when Beneviene first described them during the autopsy of a child [2].

The incidence varies from 1 per 100,000 to 250,000 admissions [3]. However, statistics are scarce, and pseudocysts represent a poorly studied pathology, which sometimes leads to the terms cyst and pseudocyst being used interchangeably and discrepancies among different authors when referring to identical concepts.

There are several types of mesenteric cysts: lymphatic cysts (simple lymphatic cyst and cystic lymphangioma), mesothelial cysts (simple mesothelial cyst, benign cystic mesothelioma, malignant cystic mesothelioma), enteric cysts (enteric duplication cysts and enteric cysts), urogenital cysts, mature cystic ovarian teratoma (dermoid cyst), and non-pancreatic pseudocysts [4].

There are three types of cyst presentation: nonspecific abdominal symptoms, incidental findings, or acute abdomen. Abdominal pain is the main symptom [1]. The typical presentation of mesenteric and omental cysts is bowel obstruction, with possible volvulus, adjacent intestinal ischemia, and acute abdominal pain. Incidental or intraoperative findings can also confirm a diagnosis other than a mesenteric cyst [5]. Mesenteric cysts can be located from the duodenum to the rectum, more frequently in the ileal mesentery of the small intestine (67%) and the mesocolon (33%). They can present serious complications such as bowel perforation, hemorrhage, and obstruction, among others. Rupture is a rare condition and usually occurs after abdominal trauma [6]. Malignant cysts account for less than 3% of cases.

The standard treatment for cysts is enucleation [1].

## Case report

**Fig. 1:**
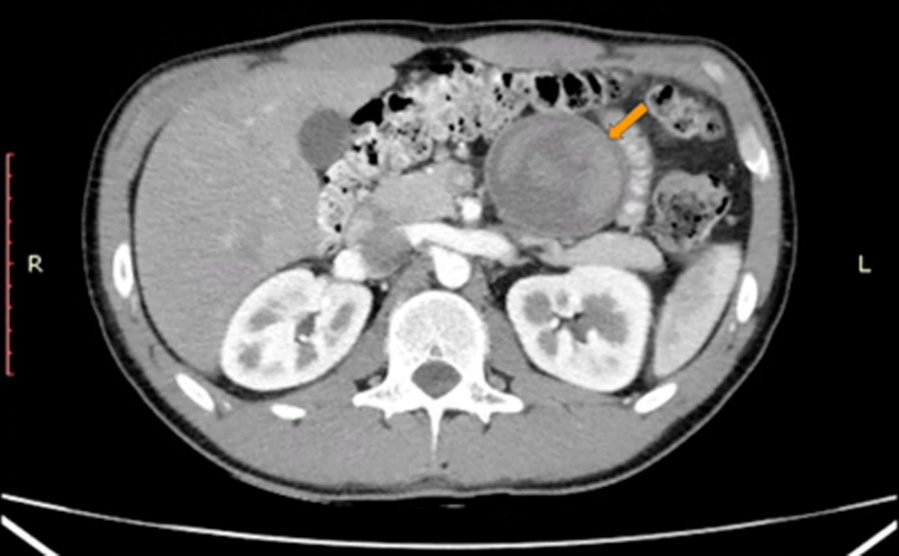
Axial section of contrast-enhanced abdominopelvic computed tomography showing a 6-cm cystic tumor with mixed density content in contact with the anterior border of the body and tail of the pancreas, small bowel, and mesenteric vessels—marked with an orange arrow

We describe the case of a 28-year-old-male patient with rib trauma who presented an incidental imaging finding in a contrast-enhanced abdominopelvic computed tomography of a 6-cm cystic appearance lesion with mixed density and hypodensity in some areas and soft-tissue density in the areas in contact with the anterior border of the pancreas (Fig. 1). The mass was also in contact with small bowel and mesenteric vessels. Potential differential diagnoses considered were solid-cystic papillary tumor or mucinous cystadenoma.

A magnetic resonance imaging (MRI) was requested, which showed a heterogeneous cystic-looking 6-cm lesion with minimal peripheral calcifications in the omental bursa in close relation with the pancreatic tail with an apparent separation plane.

**Fig. 2:**
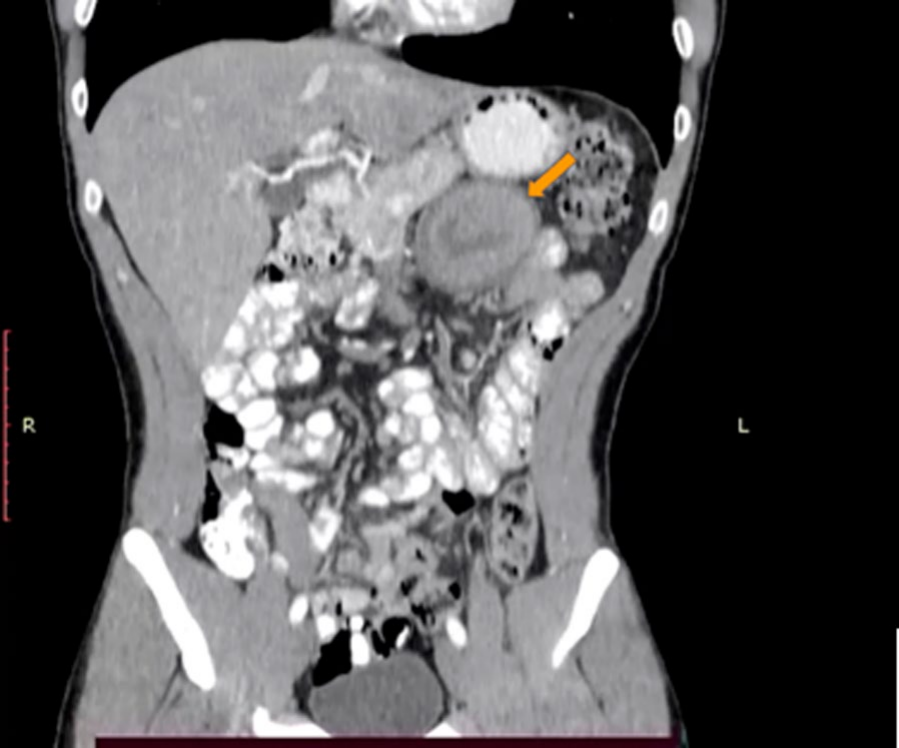
Coronal section of abdominopelvic computed tomography with double contrast, lesion marked with an orange arrow

The patient also reported a previous episode of melena (black stool), which raised the possibility of bowel duplication as differential diagnosis (Fig. 2).

**Fig. 3:**
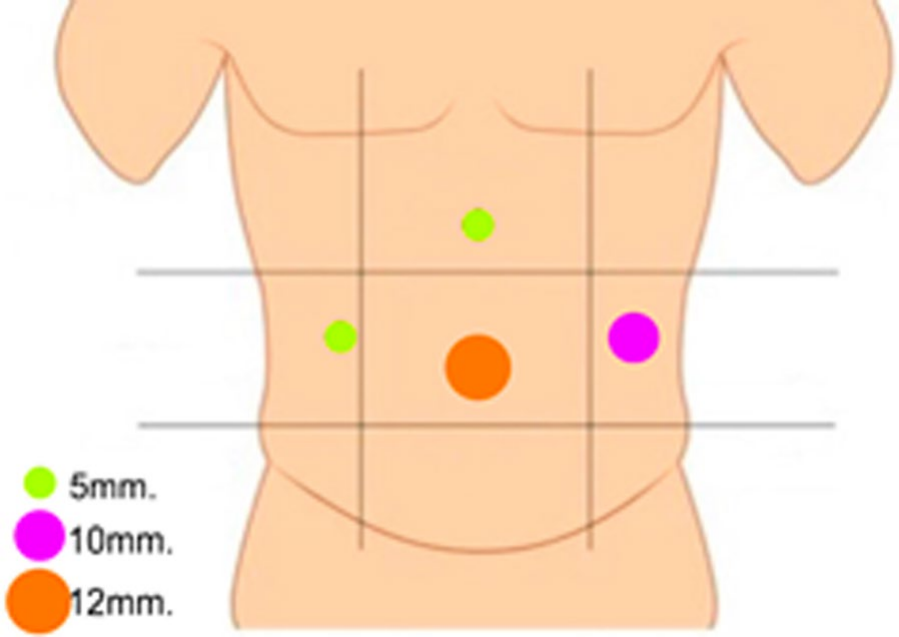
Port placement

The patient was admitted and underwent a scheduled resection surgery of a retroperitoneal cystic tumor. The patient was positioned in dorsal decubitus and the ports were placed as follows (Fig. 3).

**Fig. 4:**
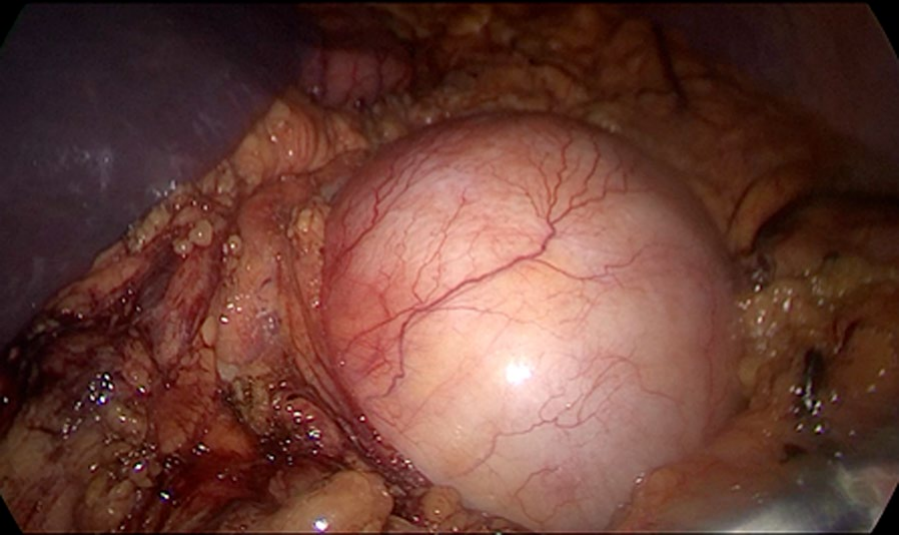
Laparoscopic image of the tumor

**Fig. 5:**
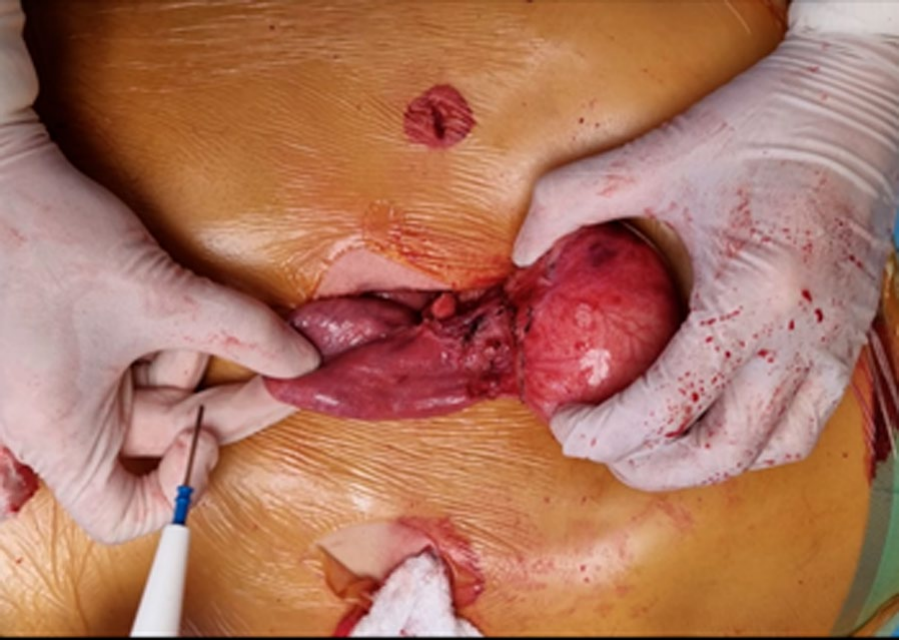
Mini-laparotomy, tumor in proximity to the first jejunal loop

**Fig. 6:**
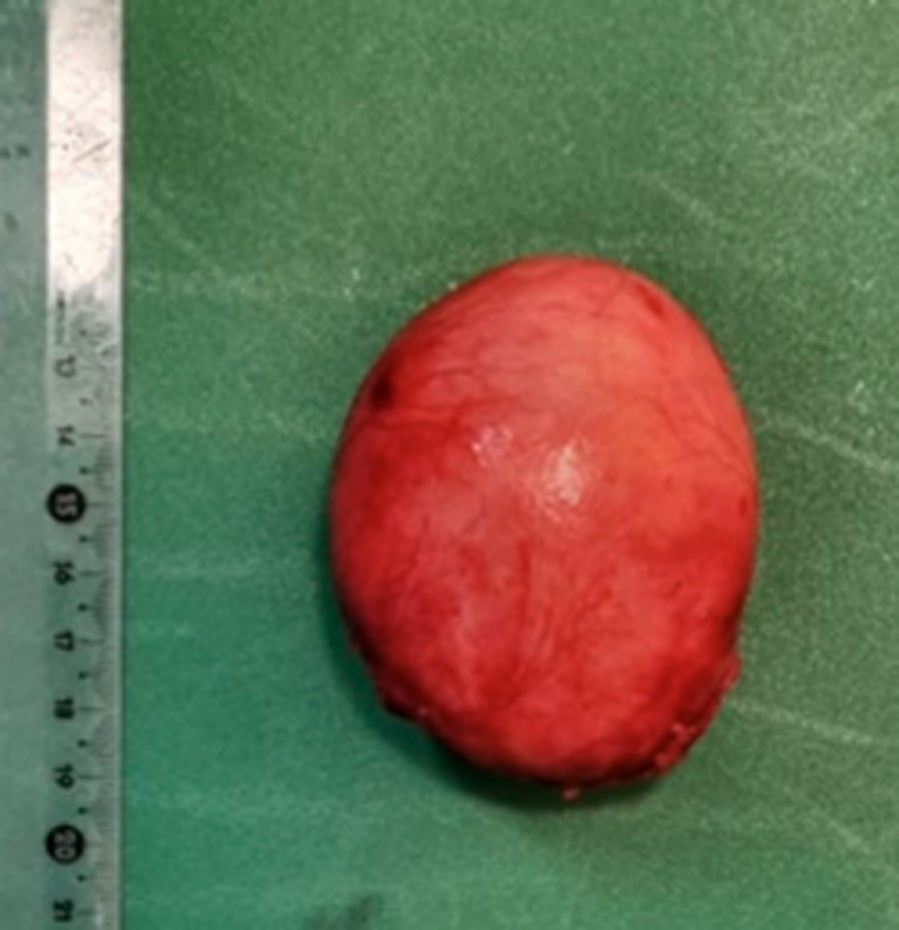
Sample

Exploratory laparoscopy did not reveal any signs of peritoneal carcinomatosis or metastasis. First, the gastrocolic ligament was dissected with a Harmonic scalpel, preserving the vascular arcade of greater gastric curvature, which enabled access to the omental bursa. At the root of the mesentery, in proximity to the first jejunal loop, an approximately 6 cm diameter, firm, elastic, rounded lesion was identified (Fig. 4). Resection was performed preserving the capsule. Due to its close proximity to the first jejunal loop and because of the impossibility of performing a safe resection, a supraumbilical midline incision was made (Fig. 5). Once the cavity was opened, a complete resection of the tumor was performed, and the sample was sent to the pathology department for further study (Fig. 6).

The patient had a favorable postoperative progress. He remained at the institution for 36 h.

**Fig. 7:**
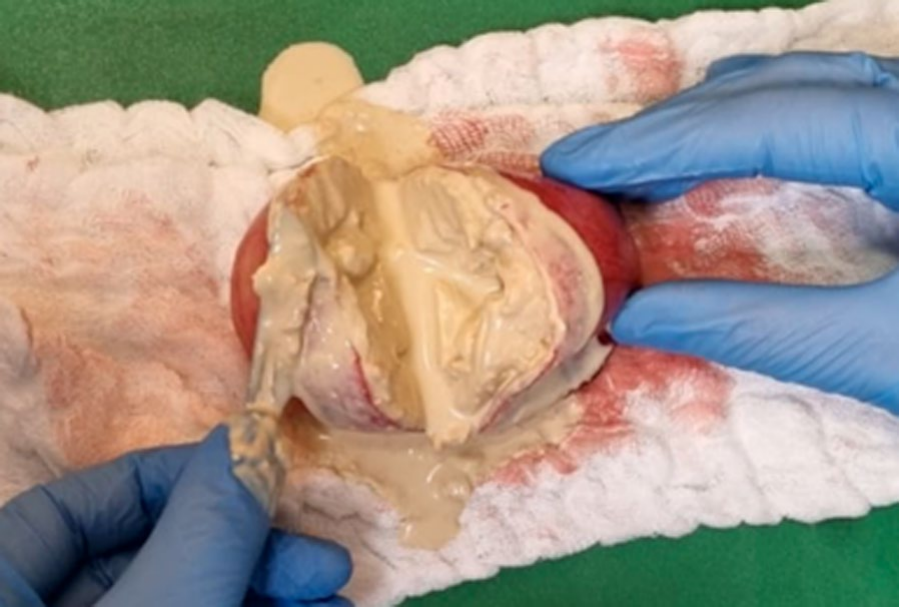
Incision on the sample showing friable, heterogeneous, whitish content

**Fig. 8:**
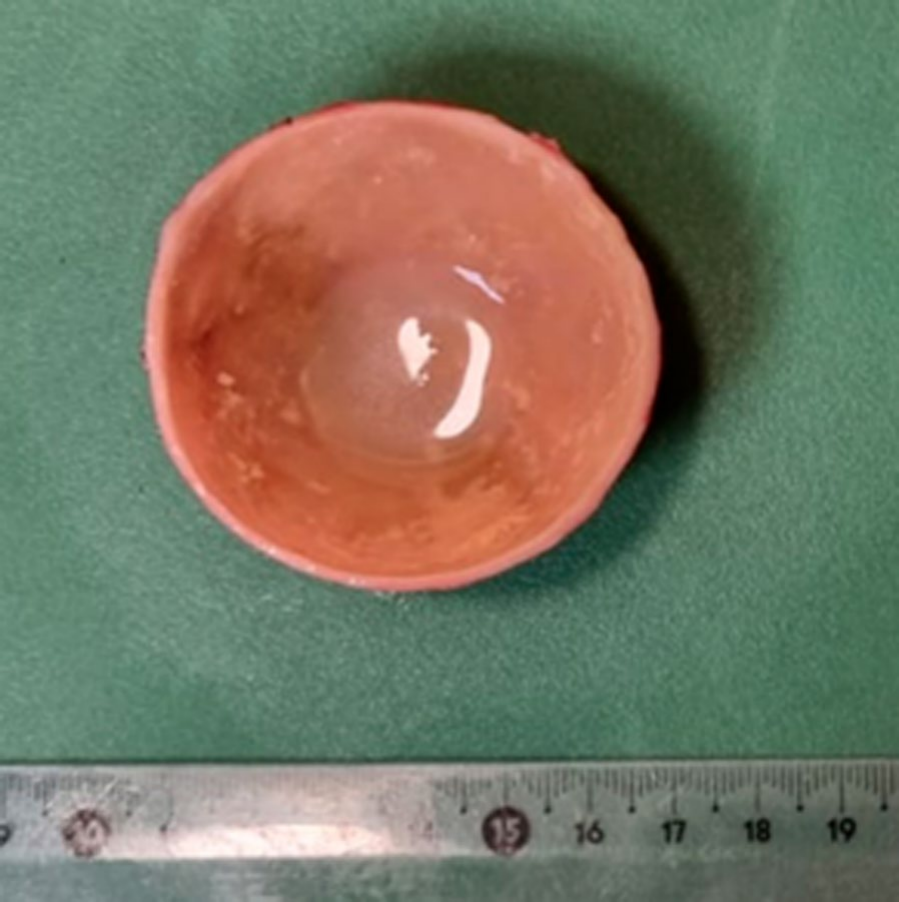
Sample. Internal walls

**Fig. 9:**
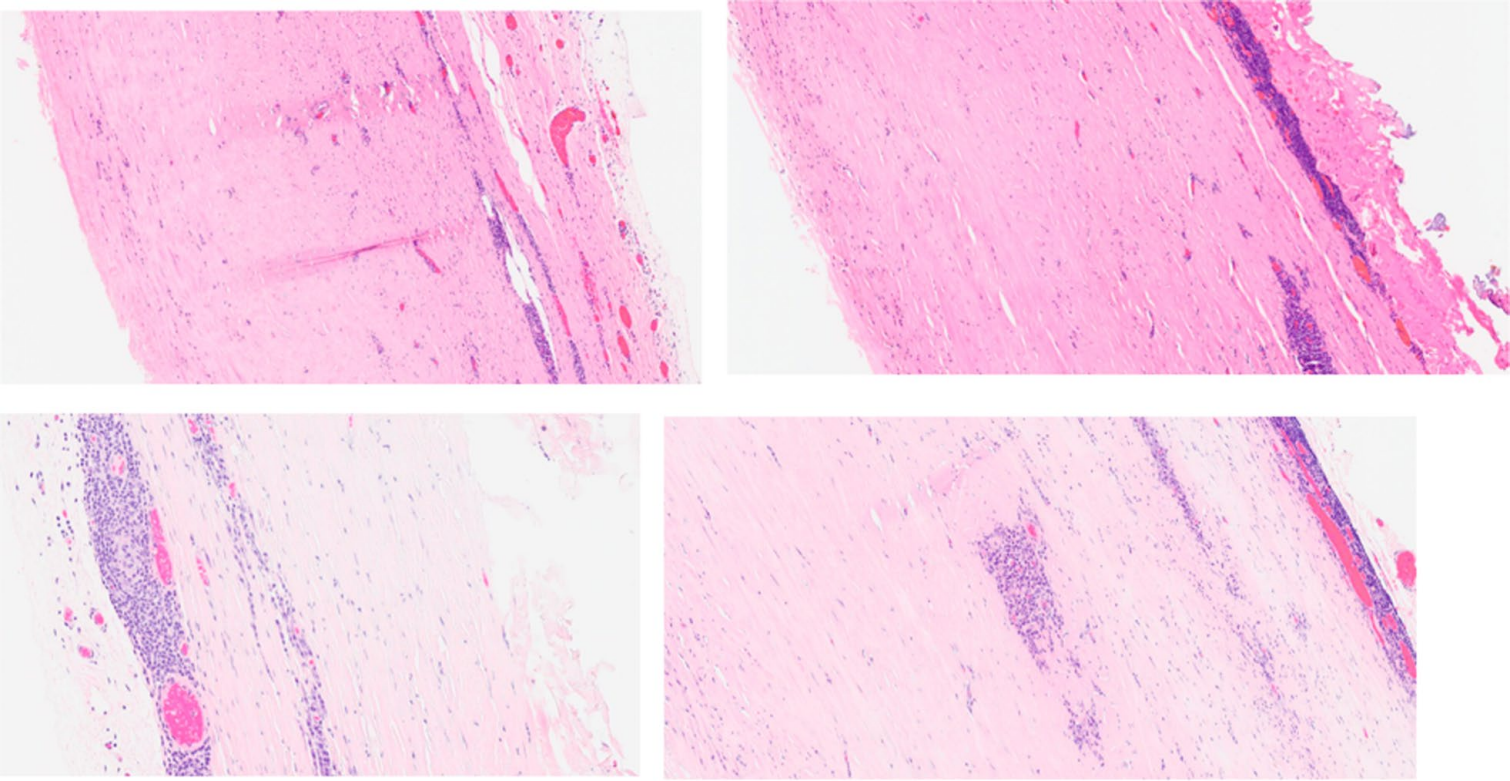
HE 100X. Histological sections showed a cyst wall formed by dense fibrous tissue. Lymphoplasmacytic inflammatory infiltrate was identified. Absence of epithelial lining

**Fig. 10:**
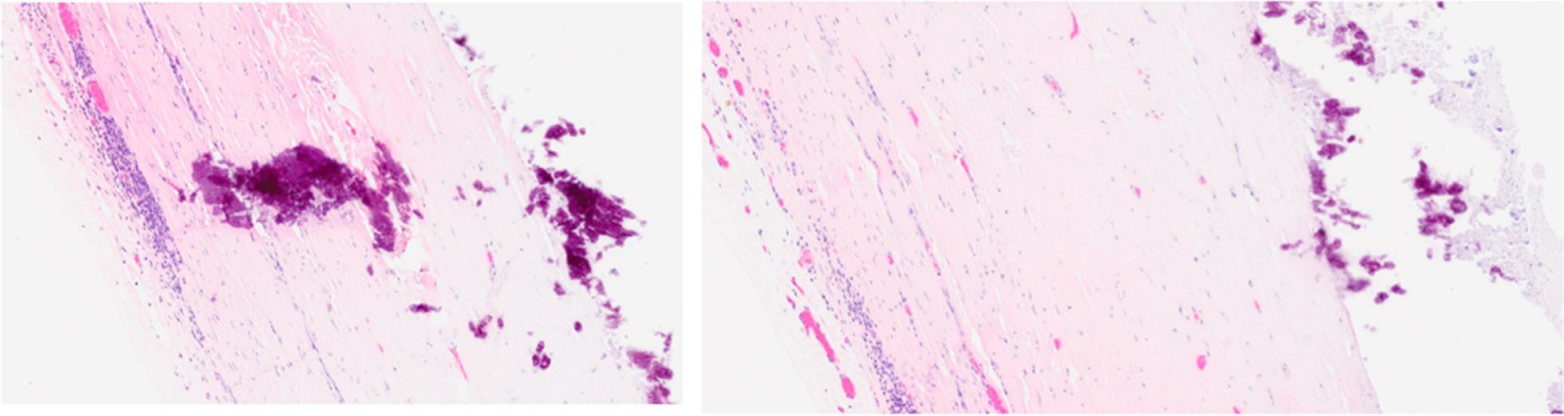
HE 100X. Histological sections showed a cyst wall formed by dense fibrous tissue. Absence of epithelial lining. Calcification foci were also identified

Macroscopically, a cystic formation of 7.6 × 6 × 4 cm, with a smooth brownish external surface (Fig. 6) was identified. Upon sectioning, it showed a smooth whitish inner wall up to 0.3 cm of thickness, with friable whitish material inside (Figs. 7, 8). The deferred anatomic pathology study, upon microscopic examination (Figs. 9, 10), showed that the walls were formed by dense fibrous tissue. No muscle tissue compatible with an intestinal duplication cyst was identified. Occasional pigmented histiocytes and partially calcified amorphous material were observed on the inner surface. No epithelial lining was identified on the inner surface. No hyaline material consistent with a hydatid cyst was observed.

The anatomic pathology diagnosis was mesenteric root pseudocyst.

## Discussion

Mesenteric cysts are one of the rarest abdominal tumors, with a reported incidence of 1 case per 100,000 to 250,000 admissions. Approximately 820 cases have been reported since 1507, when Beneviene first described them during the autopsy of a child [1, 7, 8]. They can occur at any age, with a peak incidence in the fourth decade of life [8].

The etiology is still unknown. Some authors believe they arise from the continuous growth of congenital malformations or ectopic lymphatic tissues, while others suggest that they are a developmental anomaly secondary to trauma, degeneration of lymph nodes, lymphatic vessel obstruction, or abnormal fusion of the mesentery layers. This variability in proposed etiological theories may be because it is a multifactorial phenomenon [1, 8]

Perrot et al. described a classification based on histopathological characteristics, which includes the following 6 groups: (1) lymphatic origin cysts (simple lymphatic cyst and lymphangioma); (2) mesothelial origin cysts (simple mesothelial cyst, benign cystic mesothelioma, and malignant cystic mesothelioma); (3) enteric origin cysts (enteric cyst and intestinal duplication cyst); (4) urogenital origin cysts; (5) mature cystic teratoma (dermoid cyst); and (6) non-pancreatic pseudocysts (of traumatic or infectious origin) [8].

From a pathological point of view, mesenteric cysts vary in size and shape, ranging from a few centimeters to a size that occupies most of the abdominal cavity, with sizes ranging from 2 to 35 cm [8]. They can be single or multiple, unilocular or multilocular. The content varies from serous to chylous or deep brown, depending on the location and the presence or absence of hemorrhage [1, 7]. Microscopically, these cysts have a lining of fibrous tissue or a single layer of endothelial cells. Wall calcification is unusual.

Mesenteric cysts can vary in their location, from the duodenum to the rectum. However, 50% of them are located in the small intestine, and 25% of those are in the ileal mesentery [7]. Malignant forms have been reported with an incidence of less than 3% [1], most of them were sarcomas, and some reported cases were adenocarcinomas [8].

The clinical presentation is highly variable, with no pathognomonic signs or symptoms, and is dependent on various factors: cyst size, location, and presence or absence of complications. Three forms of presentation can be described:

1. Asymptomatic (40–45%): Diagnosis is generally using imaging or surgical findings;
2. Nonspecific abdominal symptoms: This includes abdominal pain and distension, occasionally associated with nausea, vomiting, diarrhea, constipation, and weight loss;
3. Acute abdomen (30%): Arises secondary to complications of the cyst, which can include obstruction (due to compression of adjacent intestines), volvulus (which can progress to gangrene, peritonitis, and shock), hemorrhage (secondary to trauma and erosion), infection, or rupture of the cyst.

The most common symptom is abdominal pain (55–82%), followed by the presence, upon physical examination, of a palpable abdominal mass that is smooth, rounded, and compressible (55–61%), and abdominal distension (17–61%) [7, 8].

The non-specificity of the clinical presentation makes the diagnosis of a mesenteric cyst challenging. In addition, laboratory results do not contribute to the diagnosis. There are no specific radiological signs. However, ultrasound and computed tomography (CT) are considered the two most useful complementary studies [1]. Both show the cystic nature of the lesion, the absence of connection of the cystic mass with other organ structures, the presence of internal septa, and the thickness of the wall. Compared to ultrasound, CT provides greater certainty in determining the nature of the cystic content. The MRI has higher accuracy in determining the topographic location of the cyst as well as its content [8].

The standard treatment for mesenteric cysts is complete surgical enucleation. Other procedures such as aspiration and marsupialization are not recommended as they are associated with a high recurrence rate and risk of infection. In cases of malignant cysts, acceptable cure rates were reported following enucleation with clear margins. It is worth mentioning that, depending on the involvement of the neighboring structures, an intestinal resection followed by a small intestinal anastomosis may be necessary for cyst resection. The laparoscopic surgical approach should be adopted, only resorting to the conventional approach for cases with technical difficulty due to the location and/or size of the lesion.

## Conclusion

Mesenteric cysts are rare, and their asymptomatic or nonspecific symptoms often lead to diagnosis based on imaging findings in a considerable number of patients. Complete enucleation, ideally via laparoscopic approach, is the standard treatment, with a favorable long-term prognosis.

## Data Availability

Patient information: Computer System of Hospital Italiano de Buenos Aires.

## Abbreviations

MRI: Magnetic resonance imaging
CM: Centimeters
CT: Computed tomography
